# Seroprevalence of *Ehrlichia* spp., *Anaplasma* spp., *Borrelia* *burgdorferi sensu lato*, and *Dirofilaria immitis* in Stray Dogs, from 2016 to 2019, in Southern Italy

**DOI:** 10.3390/ani11010009

**Published:** 2020-12-23

**Authors:** Angela Petruccelli, Gianmarco Ferrara, Giuseppe Iovane, Rita Schettini, Roberto Ciarcia, Vincenzo Caputo, Marina Pompameo, Ugo Pagnini, Serena Montagnaro

**Affiliations:** 1 Department of Veterinary Medicine and Animal Productions, University of Naples, “Federico II”, Via Delpino 1, 80137 Naples, Italy; angela.petru@hotmail.it (A.P.); jammaferrara@hotmail.it (G.F.); giuseppe.iovane@unina.it (G.I.); roberto.ciarcia@unina.it (R.C.); upagnini@unina.it (U.P.); 2 Veterinary Service, ASL Salerno, Piazza Santini Carlo 1, 84047 Capaccio Scalo, Italy; serena.montagnaro@gmail.com; 3 Veterinary Public Health Coordination Area, Veterinary Hospital Presidium, ASL 1, Via Marco Rocco di Torrepadula, 80145 Napoli, Italy; mont.ser@virgilio.it; 4 U.O.C. Animal Health, Veterinary Hospital Presidium, ASL 1, Via Marco Rocco di Torrepadula, 80145 Napoli, Italy; marina.pompameo@virgilio.it

**Keywords:** dogs, epidemiology, canine vector-borne diseases, seroprevalence, Southern Italy

## Abstract

**Simple Summary:**

A retrospective study was carried out on presence of canine vector-borne disease (CVBD) antibodies in stray dogs in the Campania Region, from 2016 to 2019. Serum samples from stray dogs (*n* = 1023) were tested for *Ehrlichia* spp., *Anaplasma* spp., *Borrelia burgdorferi sensu lato (s.l.),* and *Dirofilaria immitis* using the SNAP^®^ 4Dx^®^ Plus Test Kit, (IDEXX Laboratories, Inc, Westbrook, ME, USA). The most common pathogen was *Ehrlichia* spp., with a percentage of positivity of 16.03%, followed by *Anaplasma* spp. with 7.8%. *B. burgdorferi s.l.* and *D. immitis* were observed in only 0.2% of animals.

**Abstract:**

Canine vector-borne diseases (CVBD) are an important and emerging health concern for humans and animals worldwide. The purpose of the presented study was to assess, from 2016 to 2019, the seroprevalence of CVBD agents and clarify the epidemiology of tick-borne disease in stray dogs living in the Campania Region, Southern Italy. For this purpose, blood samples were collected from January 2016 to December 2019 from 1023 dogs in authorized kennels located in the five municipalities of the Campania Region. SNAP^®^ 4DX^®^ from IDEXX^®^ Laboratories was used for detection of *Ehrlichia* spp., *Anaplasma* spp., *Borrelia burgdorferi sensu lato (s.l.),* and *Dirofilaria immitis* antibodies. The overall seroprevalence of CVBD in stray dogs was 19.6% (95% Confidence Intervals (CI): 17.2–22.8%; 201/1023). The most common pathogen was *Ehrlichia* spp., with a percentage of positivity of 16.03%, followed by *Anaplasma* spp. with 7.8%. *B. burgdorferi s.l.* and *D. immitis* were detected in only 0.2% of dogs; co-infection was detected in 4.5% of stray dogs tested. No link was detected between the gender, age, location, and CVBD seropositivity, except for *Ehrlichia* spp. for which location (Avellino Province; *p* = 0.007) and gender (male, *p* = 0.002) were risk factors for seropositivity. Our results demonstrated that animals are exposed to at least one of the four etiological agents (*Ehrlichia* spp., *Anaplasma* spp. *Borrelia burgdorferi s.l.*, and *Dirofilaria immitis*) transmitted by vectors. Finally, this study highlighted the utility of serological monitoring in stray dogs, housed in kennels, given the threat posed by CVBD to animals and the zoonotic implications of these etiological agents and their vectors on human health.

## 1. Introduction

In recent years, canine vector-borne diseases (CVBD) have been described as an important and emerging health concern for humans and animals worldwide. Several anthropogenic effects, such as climate change, globalization, increase in international transport and trade, and the rapid growth of the human and canine population, have caused a change in worldwide distribution of CVBD [1]. In particular, the climate can modify the distribution of vectors, their life cycle, survival, and reproduction, while the close relationship between human and pet dog can increase the risk of zoonosis [2]. CVBD can have a significant impact on the health of the dog and can also cause clinical disease in humans [3]. In this context, the control of CVBD is essential both for the health of dogs and for public health [4].

The etiology of these CVBD diseases is multiple and can involve various type of pathogens (protozoa, helminths, bacteria, and viruses) transmitted by hematophagous insects, such as ticks, fleas, lice, etc. [1,2]. The CVBD symptoms in dogs are frequently vague and nonspecific; clinical presentation may include fever and mild lameness [5,6,7,8]. Sometimes, these diseases can have severe health implications, such as nephritis in Lyme disease [9], thrombocytopenia, and/or death in *Ehrlichia* and *Anaplasma* infections [8,9]. In addition, the dogs subclinically infected can be reservoir hosts for arthropod-transmitted zoonotic pathogens [10].

*Ehrlichia canis* is a Gram-negative obligate intracellular bacterium, an etiologic agent of canine monocytic ehrlichiosis, transmitted worldwide by *Rhipicephalus sanguineus* (brown dog tick) [10,11,12]. The natural hosts of *E. canis* are dogs and other canids. Infected animals show variable clinical signs and infection may persist for many years. The disease usually becomes chronic: This stage is characterized by lethargy, thrombocytopenia, pancytopenia, weight loss, lymphadenopathy, and persistent seropositivity. Usually, ehrlichiosis is not considered a zoonotic disease, but Perez et al. (2006) [13] described some cases of human infection in Venezuela.

Anaplasmosis is a CVBD caused by bacteria belonging to the genus *Anaplasma* that infect different species, including dogs and human, and have a worldwide distribution. The genus *Anaplasma* includes obligate intracellular Gram-negative bacteria that principally infect hematopoietic cells [14]. *Anaplasma phagocytophilum* is the etiological agent of canine granulocytic anaplasmosis, characterized by the appearance of morulae in the cytoplasm of infected neutrophilic granulocytes [15], whereas *Anaplasma platys* is the causative agent of infectious canine cyclic thrombocytopenia (ICCT), and is found in platelets [16]. Ticks involved in the transmission of *A. phagocytophilum* and *A. platys* are *Ixodes* spp. and *Rhipicephalus* spp., respectively, and both are present in Italy [17].

*Borrelia burgdorferi sensu lato (s.l.)* is the aetiologic agent of Lyme disease (borreliosis), the most commonly described vector-borne disease in Europe [3]. The infection is transmitted to mammals with the bite of ticks such as *Ixodes* spp., including *Ixodes ricinus*, the main vector of the disease in European countries. Lyme disease can affect dogs, human, and potentially cats. Typical clinical signs of the disease in dogs may include fever, lethargy, anorexia, depression, generalized joint pain or arthritis, and intermittent joint lameness [18].

*Dirofilaria immitis* is a parasitic nematode that is present as an adult form in the pulmonary arteries and in the heart of infested dogs. Dirofilariasis is transmitted by mosquitoes and the domestic dog is the definitive host. The symptoms of the disease are cough and exercise of intolerance, but many dogs are subclinically infected [19]. Although there is a risk of zoonotic transmission, human heartworm infection is not common. In Europe, *D. immitis* infection occurs in the countries of the Mediterranean Basin, but the largest endemic area is located along the Po Valley in Northern Italy [20]. For a long time, the filariasis was confined in this area, but more recently this parasite has been detected in non-endemic areas of Central and Southern Italy [21,22].

The presence of CVBD agents has been described in Italy [17,23,24], but information about seroprevalence in the stray dog population is limited. Furthermore, it is difficult to extrapolate information from different studies because the prevalence of CVBD is associated with the diagnostic test used, with the geographical area, with the density of tick populations, and with the existence of reservoir species.

Thus, the aim of the presented retrospective study was to evaluate, from 2016 to 2019, the seroprevalence of CVBD agents and clarify the epidemiology of tick-borne disease in stray dogs, housed in kennels, living in the Campania Region, Southern Italy.

## 2. Materials and Methods

### 2.1. Ethical Approval

Our study did not require ethical approval. In fact, the serum samples used in our study were randomly selected from those collected from stray dogs, housed in kennels, during periodic checks (which included blood tests, screening for infectious and parasitic diseases, etc.) carried out by the government veterinary service. The residual serum from the routine tests was sent to our laboratory for our research.

### 2.2. Study Area and Sample Size

The Campania Region is located in the south of Italy, and stretches along the Tyrrhenian Sea and extends over an area of 13,595 Km2 (41°00′00″ N–14°30′00″ E). The climate is Mediterranean in the coastal areas and continental in the mountainous hinterland. The study was performed in dogs resident in the kennels of study area. The sample size was calculated using the formula suggested by Thurshfiled et al. [25] for a theoretically “infinite” population, adding the following information: expected prevalence of *E. canis* (10.5%) [18], confidence interval (CI) 95%, and desired absolute precision (5%).

The samples were collected as part of the regional plan for control and monitoring of infectious diseases in dogs housed in municipal kennels [26]. The municipal kennel hosts stray dogs; in these structures, the animals undergo veterinary clinical visits, identification, and registration procedures. After a variable period, the stray dogs are transferred to a dog shelter and destined for adoption.

Blood samples were collected during periodic checks carried out by the government veterinary service, from January 2016 to December 2019. A total of 1023 sera were collected from dogs housed in authorized kennels, placed in the five municipalities of the Campania Region: Napoli, Benevento, Avellino, Caserta, and Salerno.

In each year of the study, at least 138 dog serum samples were randomly selected. For each sample, we recorded the location and the date of collection; however, only for 927 dogs were the sex and the age documented. Animals were classified into four age classes: <3 years old (35%), >3–6 years old (29%), >6–9 years old (21%), and ≥9 years old (15%). The sample consisted of 507 (55%) male and 420 (45%) females.

### 2.3. Sample Preparation and Serological Analysis

A blood sample was collected individually from each dog during the veterinary visit for regular check-up, annual vaccination, or routine inspection. Blood was collected from the radial vein into sterile vacuum tube (Vacutainer, Becton, Dickinson and Company Franklin Lakes, NJ, USA) without anticoagulant and stored in the refrigerator for a maximum of 24 h until centrifugation. Samples were centrifuged at 1300–1800 × g for 20 min, and then serum was separated from the clot. Finally, serum were stored at −20 °C until further assayed.

To diagnose CVBD, a rapid enzyme-linked immunosorbent assay (ELISA) kit (SNAP^®^ 4Dx^®^ Plus Test Kit, IDEXX Laboratories, Inc, Westbrook, ME, USA) was used in accordance with the manufacturer’s instructions. This qualitative test allowed us to detect at the same time the presence of circulating antibodies, G and M immunoglobulins, against immunodominant proteins of *Ehrlichia* spp. (p30 and p30-1), *A. phagocytophilum* (p44/MSP2), *B. burgdorferi s.l.* (C6), and *D. immitis* (antigens principally produced by adult females).

The SNAP^®^ 4Dx^®^ Plus Test Kit showed a sensitivity and specificity of 96.2 and 100% for *E. canis*, 99.1 and 100% for *Anaplasma* spp., 98.8 and 100% for *B. burgdorferi s.l.*, and 99.2 and 100% for *D. immitis* [27]. 4Dx^®^ Plus Test Kit has been validated in dogs [27,28].

### 2.4. Statistical Analysis

Statistical analysis was performed using MedCalc Statistical Software version 16.4.3 (MedCalc Software, Ostend, Belgium; www.medcalc.org; 2016). Chi-square tests were used compare proportions of positivity related to categorical dependent variables and to establish statistical significance within each class (age, gender, year, and location).

The variables associated with seroprevalence for CVBD were applied to binary logistic models using JMP Pro version 15.0.0 (SAS Institute Inc., Campus Drive, Cary, NC, USA). *p* < 0.05 was considered significant. Significant differences between categories were quantified calculating odds ratios (OR) and their 95% confidence intervals (CI).

## 3. Results

A total of 1023 stray dogs, housed in kennels, were tested over the study period. Information about location and period of sampling was recorded for all serum samples; gender and age were recorded only for 927 animals.

The mean age was 5.62 years (>3–6 years). There were 420 males (45%) and 507 females (55%). The province of Naples supplied the largest number of samples (35%, *n* = 358), followed by Caserta (29%, *n* = 293), Avellino (17%, *n* = 179), Salerno (14%, *n* = 143), and Benevento (5%, *n* = 50). The 1023 serum samples were collected in four years: 2016 (33%, *n* = 335), 2017 (14%, *n* = 145), 2018 (26% *n* = 265), and 2019 (27% *n* = 278).

The overall seroprevalence of the four CVBD was 19.6% (201/1023, 95% CI: 17.2–22.8%) of the dogs tested (Table 1). In addition, the proportion of dogs positive to one, two, or three pathogens was 15.15% (155/1023, 95% CI: 12.95–17.35%), 4.39% (45/1023, 95% CI: 3.14–5.66%), and 0.1% (1/1023, 95% CI: 13.12013 0–0.28), respectively. No positive sample was found for all four pathogens.

*E. canis* was found to be the most common CVBD causative agent with a seroprevalence of 16.03% (164/1023, 95% CI: 13.85–18.25%) (Table 2). However, we should keep in mind that the SNAP^®^ 4Dx^®^ Plus Test Kit used in this survey detects both antibodies to *E. canis* and other *Ehrlichia* species (*E. chaffeensis, E. ewingii, E. muris*) [28], but, in Italy, infections by *E. chaffeensis, E. ewingii*, and *E. muris* are not considered important in the dog population. Thus, it is plausible to ascribe to *E. canis* the seropositivity found in our study [24].

*E. canis* seroprevalence, detected in 2016 and 2017, was statistically significant (Chi-square test: Degrees of freedom (DF) = 3, *n* = 1023, *p*-value = 0.008) compared to those observed in the other years (Table 2).

The univariate and multivariate analyses showed a statistical association between location (Chi-square test: DF = 4, *n* = 1023, *p*-value = 0.007), gender (Chi-square test: DF = 1, *n* = 927, *p*-value = 0.002), and *E. canis* seropositivity, over the 2016–2019 period (Table 2 and Table 3). A risk of *E. canis* seropositivity was significantly correlated with the kennel locations in the Avellino Province (21.23%, CI 95% 15.3–27.1) with odds ratios of 1.41 (CI 95% 0.61–3.26), 1.31 (CI 95% 0.81–2.09), 2.26 (CI 95% 1.38–3.70), and 1.01 (CI 95% 0.59–1.74), respectively, versus Benevento, Caserta, Napoli, and Salerno Provinces (Table 2).

Data analysis indicated that there was not statistical association between *E. canis* seropositivity and age (Chi-square test: DF = 3, *n* = 927, *p*-value = 0.366); however, the highest percentage of positive animals was observed in dogs aged ≥9 years old. Gender was a risk factor for *E. canis* exposure; in fact, males showed a seroprevalence of 20.24 (CI 95% 16.4–24.08) with an odds ratio of 1.78 (CI 95% 1.25–2.55) compared to females (Table 3).

*Anaplasma* spp. antibodies were detected in 7.8% (80/1023, CI 95% 6.2–9.4) of stray dogs in this study. Seropositivity to *Anaplasma* spp., in 2016, was 11.34% (CI 95% 7.94–14.74). This value was statistically significant (Chi-square test: DF = 3, *n* = 1023, *p*-value = 0.034) compared to those observed in the other years, with odds ratios of 2.19 (CI 95% 0.99–4.82), 1.99 (CI 95% 1.08–3.65), and 1.84 (CI 95% 1.02–3.31), respectively, versus 2017, 2018, and 2019 (Table 4). The univariate and multivariate analyses showed that there was not any statistically significant difference in the seroprevalence related to location (Chi-square test: DF = 4, *n* = 1023, *p*-value = 0.399), even though the highest percentage of seropositive dogs was detected in the Avellino Province (10.6%, CI 95% 6.11–15.11), followed by Caserta (8.87%, CI 95% 5.67–12.07), Napoli (6.42%, CI 95% 3.89–8.95), Salerno (6.29%, CI 95% 2.39–10.19), and Benevento (6.0%, CI 95% 0–12.5) (Table 4).

No significant difference in the seroprevalence to *Anaplasma* spp. was observed in dogs of different ages (Chi-square test: DF = 3, *n* = 927, *p*-value = 0.532) and genders (Chi-square test: DF = 1, *n* = 927, *p*-value = 0.528), although the highest seroprevalence value was detected in animals aged >3–6 years (9.29%, CI 95% 5.82–12.76) and in females (8.48%, IC 95% 0.51–1.34) (Table 5).

In view of very low seroprevalence observed in the study sample, *B. burgdorferi s.l.* (0.1%) and *D. immitis* (0.2%) were not included in the statistical analysis of the risk factors.

## 4. Discussion

CVBDs have a great scientific interest worldwide and are very important topic for both veterinary medicine and public health. The aim of this study was to evaluate the seroprevalence of CVBD in stray dogs, housed in kennels, in the Campania Region and to assess the risk factors associated with these diseases. For this purpose, a large-scale screening of 1023 stray dogs was performed during the veterinary examination for the regular check-up, annual vaccination, or routine inspection, in accordance with Regional Law of 24 November 2001, n. 16 [26].

The seroprevalence of CVBD in stray dogs was 19.6%, and the most common pathogen was *Ehrlichia* spp., with a percentage of positivity of 16.03%, followed by *Anaplasma* spp. with 7.8%. *B. burgdorferi s.l.* and *D. immitis* were detected in only 0.2% of dogs; co-infection was detected in 4.5% of the stray dogs tested (Table 1).

Selected epidemiological variables were examined to highlight possible associations with CVBD seropositivity. There was no correlation between the gender, location, and seropositivity for CVBD, with the exception of *Ehrlichia* spp. For this pathogen the location (Avellino Province) and gender (male) were risk factors for seropositivity. No significant difference in the seroprevalence of CVBD was observed in animals of different ages, but the highest seroprevalence was detected in animals aged >9 years. This result was probably attributable to a longer duration of exposure to the vector (especially *R. sanguineus)*.

Previous surveys performed in Italy have shown slightly lower CVBD seroprevalence than those found in our work (19.6%). In fact, other studies from the Italian peninsula have indicated an overall prevalence of 10.3% and 11.9% in hunting dogs in Campania and in the Tuscany Region, respectively [17,18,19,20,21,22,23,24]. Conversely, in Southern Italy (Strait of Messina), in a serological survey performed by an immunofluorescence antibody assay in kennel dogs, Pennisi et al. [29] found a seroprevalence (57%) for at least two tick-borne pathogens significantly higher than that of the other studies performed in Italy. These different results could be attributed to several factors such as the performance of the tests used, the ecology and distribution of the vectors, the dog population density, and the climatic and geographic differences between the study areas. In fact, it has been assessed by Otranto et al. [30] that CVBD risk varies between outbreaks according to these local factors.

Our overall seroprevalence was similar to that detected in Greece (21.8%) [4], but was higher than those reported in other European countries, such as Croatia (6.1%) [31], Hungary (13.0%) [32], Romania (11.3%) [33], Finland (8.5%) [3], and in Poland in which the presence of anti-CVBD antibodies was variably detected from 8.0% to 12.3% [34,35].

Finally, similar studies carried out in Spain (37.1%) [36], Albania (25.1%) [37], Portugal (68.0%) [38], and Bulgaria (64.7%) [39] have shown higher seroprevalence than those obtained in our survey in stray dogs, housed in kennels, in the Campania Region. However, it must be considered that comparing the seroprevalence values obtained in different studies and in different geographical areas should be interpreted with caution due to the different characteristics of the used diagnostic tests [40]. Moreover, the comparison of different studies can be problematic because seroprevalence for CVBD can be influenced by the level of the ticks’ infestation and irregular and partially effective prophylactic treatment schemes against ectoparasites [29].

In our study, stray dogs were more commonly exposed to *Ehrlichia* spp. compared to the other CVBD diseases tested (16.03%). Our seroprevalence about *Ehrlichia* spp. was higher than the seroprevalences obtained in hunting dogs in Campania [24] and in Tuscany [17], but also in these studies, *Ehrlichia* was the most frequent pathogen.

In other European countries, *Ehrlichia* spp. infection seems to be less common than in the Campania Region; in fact, lower seroprevalence values were found in Croatia (0.6%) [31], Hungary (0.16%) [32], Spain (5%) [36], Poland (0.26%) [35], Finland (0.3%) [3], Greece (12.5%) [4], Portugal (5%) [38], and Romania (2.1%) [33]. On the other hand, serosurveys performed in Albania and Bulgaria [37,38,39] have shown a percentage of positivity to *Ehrlichia* spp. of 20.8 and 21% respectively; these values are slightly higher than those detected in the Campania Region in our survey.

Based on our findings, the likelihood of seropositivity to *Ehrlichia* spp. was associated mainly with the location and gender, but not with the age of the dogs tested. In fact, the dogs that were living in the Avellino Province had a higher probability of being seropositive to *Ehrlichia* spp. when compared to those living in the other provinces of the Campania Region. This result may be justified by the characteristics of this area. In fact, the territory of the Avellino Province is largely mountainous, with an intricate network of hills, valleys, and wooded areas. In this scenario, stray animals can easily come into contact with vector arthropods or with wild canids and this can facilitate movement of parasites and microorganisms from domestic canids to wildlife and vice versa [41].

In addition, it is interesting to note that the seroprevalence of *Ehrlichia* spp. showed a gradual decrease during the study period of 4 years. The seroprevalence in 2015 was higher when compared to 2019 and the difference was statistically significant. This result is probably due to the application of the regional plan for the control and monitoring of diseases in kennels which provides veterinary visits for periodic checks, annual vaccinations, and treatments for infectious and/or parasitic diseases [26].

*Anaplasma* spp. was the second pathogen affecting stray dogs in the Campania Region, with a seroprevalence of 7.8%. It is important to note that the SNAP^®^ 4Dx^®^ Plus ELISA detected antibodies to *A. platys* and *A. phagocytophilum*; therefore, we describe our results as indicative of exposure to *Anaplasma* spp. in stray dogs. Indeed, due to the cross-reactions, it is very hard to discriminate *A. platys* and *A. phagocytophilum* using serological tests [8,9,10,11,12,13,14,15,16,17,18,19,20,21,22,23,24,25,26,27,28,29,30,31,32,33,34,35,36,37,38,39,40,41,42], while mixed infections are probable [43].

Our *Anaplasma* spp. seroprevalence is higher than that reported by Piantedosi et al. [24] in hunting dogs in Southern Italy (4.4%) and by Ebani et al. [17] in the Tuscany Region (4.68%), but is similar to the value reported for Hungary (7.9%) [32] and Greece (6.2%) [4]. Conversely, higher seroprevalence have been found in some Eastern European countries such as Albania (24.1%) [37], Poland (12.31%) [35], Bulgaria (30.5%) [39], and in Portugal (13%) [38], while the presence of *Anaplasma* was found to be low in Croatia (4.5%) [31], Spain (3.1%) [36], Finland (5.3%) [3], and Romania (5.5%) [33].

No statistically significant difference in the seroprevalence for *Anaplasma* spp. was observed in animals of different location, age, and gender, but a higher seroprevalence was found in animals living in the province of Avellino. This result, as already observed in *Ehrlichia* spp. infection, is probably due to the large presence of wooded areas where various wild animals such as medium and large mammals, including foxes, roe deer, and wild boars act as hosts for ticks and can play a role in the ecology of tick-borne pathogens [44].

This hypothesis is also supported by the low seroprevalence observed for *Ehrlichia* spp. and *Anaplasma* spp. in the province of Naples, characterized by a big metropolitan, strongly urbanized area. The seropositivity to *B. burgdorferi s.l.* infection found in our study in stray dogs was very low, at 0.2% (IC 95%: 0–6.3%). This value is consistent with those reported by Piantedosi et al. [24] in hunting dogs in Southern Italy (0.3%), but lower than values detected in Central Italy (1.47%) [17]. Similar prevalence was also found in other European countries such as Croatia, Hungary, and Spain (0.4% in all three county) [31,32,33,34,35,36], Greece (0.1%) [4], and Romania (0.5%) [33], while in other parts of Europe the detected seroprevalence to *B. burgdorferi s.l.* was slightly higher than that detected in our study (Poland 3.5%; Bulgaria: 2.4%; Finland: 2.9%; Portugal: 8.5%) [3,4,5,6,7,8,9,10,11,12,13,14,15,16,17,18,19,20,21,22,23,24,25,26,27,28,29,30,31,32,33,34,35,38,39]. However, we cannot exclude that the low seropositivity values may be due to the characteristics of the SNAP 4Dx. In fact, this diagnostic test detected antibodies against *B. burgdorferi s.l.* only during active infection [45].

Only two stray dogs tested were found positive to *D. immitis*, but no speculations can be made about this data because their remote case history is unknown.

## 5. Conclusions

This is the first large-scale seroprevalence investigation on stray dogs, housed in kennels, performed in Southern Italy. Unfortunately, it was not possible to investigate the role of treatments against ectoparasites due to the unknown history of infection of many animals, which were treated only after being housed in kennels. However, it is assumed that all dogs tested were infested with CVBD during their period of stray life.

Our results demonstrate that animals are exposed to at least one of the four etiological agents (*Ehrlichia* spp., *Anaplasma* spp., *B. burgdorferi s.l.,* and *D. immitis*) transmitted by vectors. Some of these pathogens are also the cause of zoonoses; therefore, control of vector-borne infections is not only of veterinary interest, but also a public health priority, as demonstrated by the reduction in the seroprevalence of *Ehrlichia* spp. from 2017 to 2019, after the application of the regional health plan.

Finally, this study highlights the utility of serological monitoring in stray dogs, given the threat posed by CVBD to animals and the zoonotic implications of these etiological agents and their vectors on human health.

## Figures and Tables

**Table 1:** Seroprevalence of canine vector-borne infections and co-infections in the stray dog population (*n* = 1023 dogs) in the Campania Region, Italy, in 2016–2019.

Pathogen	No. of Infected Dogs	Prevalence (%)
*Ehrlichia* spp.	118	11.53
*Anaplasma* spp.	36	3.51
*Borrelia burgdorferi s.l.*	1	0.1
*Dirofilaria immitis*	0	0
*Ehrlichia* spp. + *Anaplasma* spp.	43	4.2
*Ehrlichia* spp. + *Dirofilaria immitis*	2	0.2
*Ehrlichia* spp. + *Anaplasma* spp. + *Borrelia burgdorferi s.l.*	1	0.1
Overall seroprevalence	201	19.64

**Table 2:** Seroprevalence of infection with *Ehrlichia* spp. and risk factor analysis by region and year in stray dogs in the Campania Region in 2016–2019 as detected by qualitative dot-ELISA SNAP 4DX ^®^ (IDEXX Laboratories).

Factor	*N*	Positive	%	SE % ^§^	95%CI *	χ^2^	*p*-value	OR ^#^	95%CI *
Total	1023	164	16.03	0.22	13.85–18.25	−	−	−	−
Year									
2016	335	66	19.70	4.20	15.5–23.9	11.92	0.008	Ref ^$^	
2017	145	30	20.69	6.6	14.11–27.27			0.94	0.57–1.52
2018	265	38	14.34	4.2	10.14–18.54			1.46	0.94–2.26
2019	278	30	10.79	3.6	10.14–18.54			2.02	1.27–3.22
Province									
Avellino	179	38	21.23	5.9	15.33–27.13			Ref ^$^	
Benevento	50	8	13.00	9.3	3.7–22.3	14.229	0.007	1.41	0.61–3.26
Caserta	293	50	17.06	4.3	13.3–21.9			1.31	0.81–2.09
Napoli	358	38	10.61	3.2	7.41–13.81			2.26	1.38–3.70
Salerno	143	30	20.98	6.7	14.28–27.68			1.01	0.59–1.74

^§^ SE: Standard error. * 95% CI: 95% confidence interval. ^#^ OR: Odds ratio. ^$^ Ref: Reference category.

**Table 3:** Seroprevalence of infection with *Ehrlichia* spp. and risk factor analysis by age and gender in stray dogs in the Campania Region in 2016–2019 as detected by qualitative dot-ELISA SNAP 4DX ^®^ (IDEXX Laboratories).

Factor	*N*	Positive	%	SE % ^§^	95%CI *	χ^2^	*p*-value	OR ^#^	95%CI *
Total	927	148	15.97	1.73	6.14–9.61	−	−	−	−
Age									
<3	320	44	13.75	3.77	9.98–17.52			Ref ^$^	
>3–6	269	48	17.84	4.58	13.26–22.42			0.73	0.47–1.14
>6–9	197	29	14.72	4.95	9.77–19,67	3.171	0.366	0.92	0.55–1.53
≥9	141	27	19.15	6.49	12.66–25.46			0.67	0.39–1.13
Gender									
Male	420	85	20.24	3.84	16.4–24.08	9.875	0.002	1.78	1.25–2.55
Female	507	63	12.43	2.87	9.56–15.3				

^§^ SE: Standard error. * 95% CI: 95% confidence interval. ^#^ OR: Odds ratio. ^$^ Ref: Reference category.

**Table 4:** Seroprevalence of infection with *Anaplasma* spp. and risk factor analysis by region and year in stray dogs in the Campania Region in 2016–2019 as detected by qualitative dot-ELISA SNAP 4DX ^®^ (IDEXX Laboratories).

Factor	*N*	Positive	%	SE % ^§^	95%CI *	χ^2^	*p*-value	OR ^#^	95%CI *
Total	1023	80	7.8	1.6	6.2–9.4	−	−	−	−
Year									
2016	335	38	11.34	3.4	7.94–14.74	8.701	0.034	Ref ^$^	
2017	145	8	5.52	3.72	1.8–9,24			2.19	0.99–4.82
2018	265	16	6.04	2.87	3.17–8.91			1.99	1.08–3.65
2019	278	18	6.47	2.89	3.58–9.36			1.84	1.02–3.31
Province									
Avellino	179	19	10.61	4.5	6.11–15.11			Ref.	
Benevento	50	3	6.00	6.5	0–12.5	4.049	0.399	1.86	0.52–6.56
Caserta	293	26	8.87	3.2	5.67–12.07			1.21	0.65–2.27
Napoli	358	23	6.42	2.53	3.89–8.95			1.72	0.91–3.26
Salerno	143	9	6.29	3.9	2.39–10.19			1.76	0.77–4.03

^§^ SE: Standard error. * 95% CI: 95% confidence interval. ^#^ OR: Odds ratio. ^$^ Ref: Reference category.

**Table 5:** Seroprevalence of infection with *Anaplasma* spp. and risk factor analysis by age and gender in stray dogs in the Campania Region in 2016–2019 as detected by qualitative dot-ELISA SNAP 4DX ^®^ (IDEXX Laboratories).

Factor	*N*	Positive	%	SE % ^§^	95%CI *	χ^2^	*p*-value	OR ^#^	95%CI *
Total	927	73	7.87	1.73	6.14–9.61	−	−	−	−
Age									
<3	320	26	8.13	2.99	11.12–11.12			Ref	
>3–6	269	25	9.29	3.47	5.82–12.76			1.15	0.65–2.05
>6–9	197	11	5.58	3.21	2.37–2.37	2.2	0.532	0.66	0.32–1.38
≥9	141	11	7.80	4.43	3.37–12.23			0.95	0.45–1.99
Gender									
Male	420	30	7.14	2.46	4.68–9.6	0.398	0.528	0.83	0.51–1.34
Female	507	43	8.48	2.43	6.05–10.91				

^§^ SE: Standard error. * 95% CI: 95% confidence interval. ^#^ OR: Odds ratio. ^$^ Ref: Reference category.

## Data Availability

Data is contained within the article.
